# Cost-Effectiveness of Thrombectomy With or Without Alteplase in Large Vessel Occlusion Stroke: A Meta-Analysis Considering Time-to-Treatment

**DOI:** 10.1212/WNL.0000000000214866

**Published:** 2026-04-17

**Authors:** Chi Phuong Nguyen, Fabiano Cavalcante, Durk-Jouke van der Zee, Kilian M. Treurniet, Manon Kappelhof, Wenjie Zi, Raul G. Nogueira, Jianmin Liu, Pengfei Yang, Kentaro Suzuki, Kazumi Kimura, Urs Fischer, Johannes Kaesmacher, Jan Gralla, Hester F. Lingsma, Yohanna Kusuma, Conor Houlihan, Peter J. Mitchell, Bernard Yan, Yvo Roos, E. Buskens, Charles B. Majoie, Maarten Uyttenboogaart, Maarten M.H. Lahr, for the IRIS collaborators

**Affiliations:** 1 Department of Epidemiology, University of Groningen, University Medical Center Groningen, Netherlands;; 2 Faculty of Pharmaceutical Management and Economic, Hanoi University of Pharmacy, Vietnam;; 3 Department of Radiology and Nuclear Medicine, Amsterdam University Medical Centers, University of Amsterdam, Netherlands;; 4 Department of Operations, Faculty of Economics and Business, University of Groningen, Netherlands;; 5 Department of Neurology, Second Affiliated Hospital of Army Medical University (Xinqiao Hospital), Chongqing, China;; 6 Department of Neurology and Neurosurgery, UPMC Stroke Institute, University of Pittsburgh School of Medicine, PA;; 7 Department of Neurosurgery, Changhai Hospital, Naval Medical University, Shanghai, China;; 8 Oriental Pan-Vascular Devices Innovations College, University of Shanghai for Science and Technology, China;; 9 Department of Neurology, Nippon Medical School, Tokyo, Japan;; 10 Department of Neurology, University Hospital Bern, University of Bern, Switzerland;; 11 Department of Neuroradiology, University Hospital Bern, University of Bern, Switzerland;; 12 Department of Public Health, Erasmus Medical Center, Rotterdam, Netherlands;; 13 School of Medicine, Deakin University, Waurn Ponds, Australia;; 14 Faculty of Health and Dentistry, The University of Melbourne, Australia;; 15 Department of Neurology, Melbourne Brain Centre, The Royal Melbourne Hospital, Australia;; 16 Department of Neurology, Airlangga University, Jakarta, Indonesia;; 17 Department of Radiology, The Royal Melbourne Hospital, University of Melbourne, Parkville, Australia;; 18 Department of Neurology, Melbourne Brain Centre, The Royal Melbourne Hospital, University of Melbourne, Parkville, Australia;; 19 Department of Neurology, Amsterdam University Medical Centers, University of Amsterdam, Netherlands;; 20 Aletta Jacobs School of Public Health, University of Groningen, Netherlands;; 21 Department of Neurology, University of Groningen, University Medical Center Groningen, Netherlands; and; 22 Department of Radiology, Medical Imaging Center, University of Groningen, University Medical Center Groningen, Netherlands.

## Abstract

**Background and Objectives:**

In stroke patients directly admitted to thrombectomy-capable centers, the value of intravenous thrombolysis (IVT) with alteplase before thrombectomy is time dependent. While early IVT may improve outcomes, delayed IVT administration lowers the likelihood of benefit. To date, no previous cost-effectiveness study has considered onset-to-treatment time. This study evaluated the cost-effectiveness of intravenous (IV) alteplase plus thrombectomy vs thrombectomy alone in patients admitted directly to thrombectomy-capable centers across 16 countries, stratified by onset-to-IVT time.

**Methods:**

A decision tree integrated with a Markov model estimated costs, quality-adjusted life years (QALYs), and incremental net monetary benefit (INMB) over 15 years. A willingness-to-pay threshold of one gross domestic product per capita was applied for each country. Effectiveness data were derived from individual patient data from 6 trials including patients with anterior circulation large-vessel occlusion eligible for both IVT and thrombectomy who presented directly to thrombectomy-capable centers. Costs were obtained from a literature review. Onset-to-IVT time was categorized as <140, 140–169, 170–199, and ≥200 minutes. One-way sensitivity and probabilistic sensitivity analyses were performed to check robustness of results.

**Results:**

Ninety-day functional outcome distributions from 2,268 patients (median age 71 years; 44% female) were used to model cost-effectiveness in a hypothetical cohort of 10,000 patients. Without accounting for onset-to-IVT time, IV alteplase plus thrombectomy seemed cost-effective in 13 countries (INMB: $85–$3,618; 50–65% probability of cost-effectiveness) and not cost-effective in the United States, China, and Vietnam, with modest health gains (0.06–0.08 QALYs per patient). Time-stratified analyses revealed that IVT plus thrombectomy was cost-effective in 16 countries when onset-to-IVT time was <140 minutes (INMB: $615–$30,645; 82%–98% probability) and at 140–169 minutes (INMB: $86–$16,918; 51%–77% probability). However, IV alteplase plus thrombectomy was no longer cost-effective in 8 countries at 170–199 minutes. Universally, the INMB was negative for onset-to-IVT times exceeding 200 minutes.

**Discussion:**

Cost-effectiveness of IV alteplase plus thrombectomy varies per country and onset-to-IVT time. IV alteplase plus thrombectomy is cost-effective when IVT can be administered within 170 minutes from symptom onset. Cost-effectiveness of IV alteplase plus thrombectomy diminishes progressively with longer onset-to-IVT times and becomes detrimental after 200 minutes.

## Introduction

Endovascular thrombectomy (EVT) has become the standard of care for patients with acute ischemic stroke due to large vessel occlusion (LVO). However, the role of intravenous thrombolysis (IVT) with alteplase before EVT remains a topic of ongoing investigation. A pooled individual participant data meta-analysis of 6 trials did not establish overall noninferiority of EVT alone compared with combined IVT plus EVT in patients with LVO who presented directly to EVT-capable stroke centers.^1^ It is important to note that a recent analysis indicated that the clinical benefit of administering IVT plus EVT was time dependent, with statistically significant advantages observed only when IVT was given early (i.e., within 140 minutes) after symptom onset.^2^

In addition to clinical effectiveness, treatment decisions in acute stroke care are influenced by economic considerations and the availability of health care resources as well. Administering IVT before EVT involves additional upfront costs while stroke-related disability can lead to significant long-term health care expenditures. The choice between IVT plus EVT and EVT alone may vary depending on local costs and system-level capacity.

Although previous cost-effectiveness studies compared intravenous (IV) alteplase before EVT and EVT alone, most have not accounted for the time-dependent nature of treatment benefits, which is crucial for accurately informing policy and clinical practice across diverse health care settings.^3-6^ Therefore, we conducted a comprehensive economic evaluation to assess the long-term cost-effectiveness of IV alteplase plus EVT vs EVT alone. Our analysis incorporates time-to-treatment interactions using data from individual participant-data meta-analysis and considers variations in health care resource utilization across different health system contexts.

## Methods

### Study Design and Participants

A model-based cost-effectiveness study was conducted to compare the costs and effectiveness of IV alteplase plus EVT vs EVT alone in patients with LVO directly admitted to EVT-capable stroke centers within 4.5 hours of symptom onset. Treatment effectiveness at 90 days was obtained from the previously mentioned individual patient-level data meta-analysis of the IRIS collaboration.^2^ In this study, we excluded 25 patients who received tenecteplase and restricted the analysis to those treated with IV alteplase in the IRIS cohort. The design and primary findings of the IRIS meta-analysis have been previously reported.^1^ The IRIS study included patients from 6 trials across 15 countries (DIRECT-MT, MR CLEAN-NO IV, SKIP, DEVT, SWIFT-DIRECT, and DIRECT-SAFE).^7-12^ A hypothetical cohort of 10,000 patients with LVO was generated, with baseline characteristics based on the IRIS population. The cost-effectiveness analysis was performed separately in 15 IRIS countries and the United States to provide a comprehensive evaluation across different settings.

### Model Description

A decision tree combined with a Markov model was developed to estimate total costs, total quality-adjusted life years (QALYs), and incremental net monetary benefit (INMB) over a 15-year time horizon. A 15-year time horizon was selected to approximate a lifetime horizon for the target population. Among 16 countries, Japan has the highest life expectancy at around 85 years, while most others range between 80 and 84 years. The decision tree captured patient pathways during the first 90 days after acute ischemic stroke and up to 1 year. Thereafter, survivors at 1 year transitioned into the Markov model with simulated health states over the subsequent 14 years. Health states in the model were defined by the modified Rankin Scale (mRS) scores (0–6), with higher scores indicating more severe disability and mRS 6 representing death.

**Figure 1:**
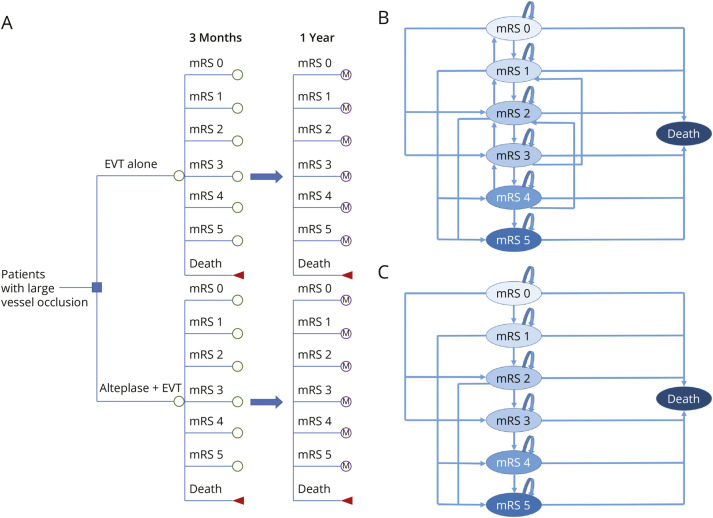
Cost-Effectiveness Model (A) Decision tree model; (B) Markov model during first 4 years; (C) Markov model after 5 years. EVT = endovascular thrombectomy; M = Markov model; mRS = modified Rankin Scale.

Patients receiving EVT alone or in combination with IV alteplase were entered according to their respective mRS health states at 90 days after stroke, based on data from 6 trials. The decision tree determined health state distributions, costs, and QALYs during the first year, while the Markov model extrapolated these outcomes from years 2 to 15. In the Markov model, during the first 4 years, patients could experience improvement, remain stable, or deteriorate in their mRS score. Beyond the fifth year, transitions were limited to remaining stable or further deterioration. We assumed that health outcomes beyond 90 days were independent of the initial treatment strategy but conditional on the health state achieved at 90 days. Figure 1 illustrates the overall model structure and transition pathways.

### Outcomes

Health economics modeling was used to estimate total costs, total QALYs, and INMB for reperfusion treatments. QALYs were defined as life years gained multiplied by corresponding health-related quality-of-life weights. The INMB was calculated using the following formula:

INMB=(WTP×incremental QALYs) – incremental costs,

where the willingness-to-pay (WTP) threshold was set at one gross domestic product (GDP) per capita for each country.^13^ Although the WHO recommends a threshold range of 1–3 times GDP per capita, a recent systematic review suggests a more appropriate range is 0.5–1.5 times GDP per capita.^14^ IV alteplase plus EVT was considered cost-effective if the INMB was positive.

### Input Parameters

#### mRS and Transition Probabilities

Patient outcomes (mRS scores) at 90 days after reperfusion treatment were obtained from individual patient data from 6 trials (Table 1).^7-12^ Time from symptom onset to IVT administration was estimated by adding the respective trial's mean time from randomization to IVT administration to the individual patient's time from symptom onset to randomization. We used the same methodology as the one used in a previous study.^2^ To estimate the distribution of mRS outcomes across onset-to-IVT time windows for the cost-effectiveness model, we applied a one-stage, pooled individual patient data approach. Additional descriptive statistics, including median (IQR) mRS by time window and median (IQR) onset-to-treatment time stratified by mRS category, are summarized in eTables 1 and 2.

**Table 1:** mRS Scores at 90 Days

	<140 min		140–169 min		170–199 min		≥200 min		All	
	N	%	N	%	N	%	N	%	N	%
EVT alone										
mRS 0	68	13	28	14	24	15	23	9	143	12
mRS 1	100	18	39	20	23	14	39	15	201	18
mRS 2	114	21	32	17	28	18	46	18	220	19
mRS 3	76	14	20	10	26	16	53	21	175	15
mRS 4	56	10	22	11	14	9	26	10	118	10
mRS 5	55	10	23	12	15	9	19	7	112	10
mRS 6	72	14	31	16	30	19	49	20	182	16
Total	541	100	195	100	160	100	255	100	1,151	100
Alteplase+EVT										
mRS 0	78	17	21	12	31	17	32	11	162	14
mRS 1	82	17	37	21	28	16	30	10	177	16
mRS 2	115	24	38	21	29	16	44	15	226	20
mRS 3	60	13	25	14	24	13	43	15	152	14
mRS 4	38	8	14	8	25	14	44	15	121	11
mRS 5	39	8	16	9	17	9	42	15	114	10
mRS 6	59	13	26	15	26	15	54	19	165	15
Total	471	100	177	100	180	100	289	100	1,117	100

Abbreviations: EVT = endovascular thrombectomy; mRS = modified Rankin Scale.

Transition probabilities of mRS scores from 90 days to 1 year and for subsequent annual cycles in the Markov model were derived from the 5-year follow-up data of the Oxford Vascular Study (eTables 3–5).^15^ Mortality over the 15-year model horizon was assumed to be dependent on both age and mRS score. Age-specific mortality risks were adjusted using country-specific life tables (eTable 6).

#### Utilities and Costs

Health-related quality-of-life weights, expressed in utility and costs, were identified through a targeted literature search (eMethods 1, eTable 7). Priority was given to country-specific data. If the data were unavailable, we used pooled estimates of utilities by mRS scores from a published meta-analysis (eTable 8).^16^ Where direct cost data were missing, costs were estimated by adapting data from comparable countries using purchasing power parity adjustments.^17^ Costs were obtained from the health care payer perspective, including costs of IVT with alteplase, costs of EVT, and costs after stroke by mRS score (eTable 9). Indirect costs (i.e., informal care and productivity loss) were not included in the study because of the lack of data. Annual discount rates for costs and QALYs were based on health economics guidelines of each country (eTable 10). All costs were converted to the year of 2023 using consumer price index, and those reported in local currency were converted to $ (USD) (eTables 11, 12).

### Statistical Analysis

#### Base-Case Analysis and Subgroup Analysis

The base-case analysis compared IV alteplase plus EVT with EVT alone across 16 countries, assuming average time to treatment. To explore the influence of treatment delays on cost-effectiveness, a subgroup analysis was conducted based on onset-to-IVT time, stratified into 4 intervals: <140 minutes, 140–169 minutes, 170–199 minutes, and ≥200 minutes. Time-to-treatment strata were defined beginning at 140 minutes of onset-to-IVT time, consistent with the previous publication,^2^ which identified this as the threshold beyond which the clinical benefit of IVT plus EVT compared with EVT alone was no longer statistically significant.

#### Sensitivity Analyses

Deterministic one-way sensitivity analyses were performed by varying input parameters by ±20% to examine their individual impact on INMB. The robustness of results was further assessed in probabilistic sensitivity analyses using Monte Carlo simulations with 10,000 iterations. In these simulations, input parameters were varied according to their respective distributions (eMethods 2 and eTable 13). We presented uncertainty as the probability that IV alteplase plus EVT was cost-effective at the threshold of one GDP per capita and across a range of WTP thresholds. The study adhered to the Consolidated Health Economic Evaluation Reporting Standards 2022 (CHEERS 2022).^18^ All analyses were performed using R version 4.1.2 and Treeage Pro 2022 R1.2.

### Model Validation

Model validation was assessed across 4 domains—conceptual model, input data, computerized model, and operational performance—using the Assessment of the Validation Status of Health Economic Decision Models tool (eTable 14).^19^ In addition, the model was also cross-validated by comparing the model's outputs with findings from previous studies.^3-6,20-23^

### Standard Protocol Approvals, Registrations, and Patient Consents

Because this study involved a model-based analysis using previously published input parameters, ethics approval was not required. The protocol was approved by the IRIS executive committee, and the study was not registered.

### Data Availability

Deidentified participant data can be made available on reasonable request through email to the corresponding author. All proposals will need to be reviewed and approved by the IRIS executive committee. Other relevant data are provided in the article and its supplementary material.

## Results

We modeled a hypothetical cohort of 10,000 patients using 90-day functional outcome distributions derived from 2,268 patients (median age 71 years; 44% female). When onset-to-IVT time was not considered, IVT with alteplase plus EVT was associated with higher costs ($469-$10,200 per patient) and modest gains in QALYs (0.06–0.08 QALYs per patient) compared with EVT alone, resulting in a positive INMB at the WTP threshold of one GDP per capita in 13 countries. The probability of IV alteplase plus EVT being cost-effective ranged from 50% to 65% in European countries and other high-income countries, including Canada, Japan, Australia, and New Zealand. By contrast, in the United States, China, and Vietnam, IV alteplase plus EVT resulted in negative INMBs and had a low probability of being cost-effective (19%–28%) at the WTP threshold of one GDP per capita (Table 2). At a higher WTP threshold of 3 times GDP per capita, IVT plus EVT was cost-effective in China (INMB $611) and Vietnam (INMB $360). However, IV alteplase plus EVT remained not cost-effective when applying the commonly used WTP threshold of $100,000 in the United States (eAppendix 1 and eTable 15).

**Table 2:** Cost-Effectiveness of IV Alteplase Plus EVT vs EVT Alone

Treatment	Total costs ($)	Incremental cost ($)	Total QALYs	Incremental QALY	INMB ($)^a^	% Of being cost-effective
Australia						
EVT alone	47,641		3.79			
IV alteplase+EVT	50,443	2,802	3.85	0.07	1,481	58%
Austria						
EVT alone	93,963		3.48			
IV alteplase+EVT	95,224	1,376	3.55	0.06	2,366	62%
Belgium						
EVT alone	149,444		3.73			
IV alteplase+EVT	151,434	1,990	3.80	0.07	1,705	59%
Canada						
EVT alone	154,501		4.17			
IV alteplase+EVT	158,118	3,617	4.24	0.08	522	53%
China						
EVT alone	18,959		3.32			
IV alteplase+EVT	20,916	1,956	3.39	0.07	−1,101	19%
Finland						
EVT alone	368,014		3.82			
IV alteplase+EVT	371,740	3,726	3.89	0.07	85	50%
France						
EVT alone	171,841		4.25			
IV alteplase+EVT	173,962	2,121	4.32	0.08	1,240	57%
Japan						
EVT alone	61,315		4.22			
IV alteplase+EVT	62,295	1,280	4.30	0.07	1,168	61%
Germany						
EVT alone	94,710		4.14			
IV alteplase+EVT	96,827	2,118	4.21	0.07	1,691	59%
Netherlands						
EVT alone	110,091		4.66			
IV alteplase+EVT	111,861	1,770	4.75	0.08	3,455	65%
New Zealand						
EVT alone	49,394		3.97			
IV alteplase+EVT	51,422	2,028	4.05	0.07	1,475	60%
Spain						
EVT alone	123,479		4.07			
IV alteplase+EVT	124,843	1,364	4.15	0.07	1,021	56%
Switzerland						
EVT alone	345,339		4.10			
IV alteplase+EVT	350,793	5,454	4.17	0.07	1,854	55%
United Kingdom						
EVT alone	87,045		3.75			
IV alteplase+EVT	89,170	2,125	3.82	0.07	1,310	58%
United States						
EVT alone	140,507		3.49			
IV alteplase+EVT	150,708	10,200	3.56	0.07	**−**4,607	28%
Vietnam						
EVT alone	7,147		2.97			
IV alteplase+EVT	7,616	469	3.03	0.06	−192	28%

Abbreviations: EVT = endovascular thrombectomy; INMB = incremental net monetary benefit; IV = intravenous; QALYs = quality adjusted-life years.

a The willingness-to-pay threshold was one GDP per capita.

**Figure 2:**
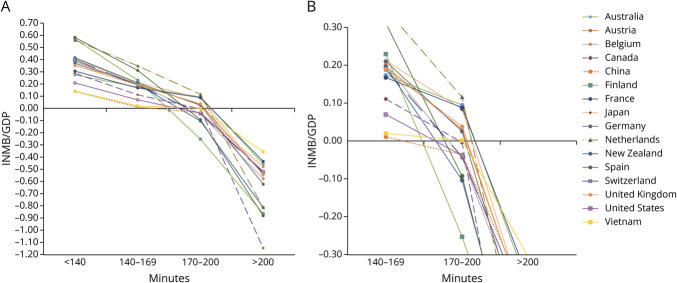
(A) INMB/GDP of IV Alteplase Plus EVT Stratified by Time-to-Treatment in 16 Countries; (B) Magnified View of INMB/GDP of IV Alteplase Plus EVT Between 170 and 200 Minutes in 16 Countries EVT = endovascular thrombectomy; GDP = gross domestic product; INMB = incremental net monetary benefit; IV = intravenous.

Including time from stroke onset to IVT in the analysis showed that IV alteplase plus EVT was cost-effective in 16 countries when treatment was initiated within 170 minutes. Beyond this threshold, the cost-effectiveness of IV alteplase plus EVT declined, and IVT plus EVT was no longer cost-effective in 8 countries, including Belgium, Canada, China, Finland, France, Spain, Switzerland, and the United States. When onset to IVT time exceeded 200 minutes, IV alteplase plus EVT was no longer cost-effective in any of the countries (Figure 2, eTables 16–19).

**Figure 3:**
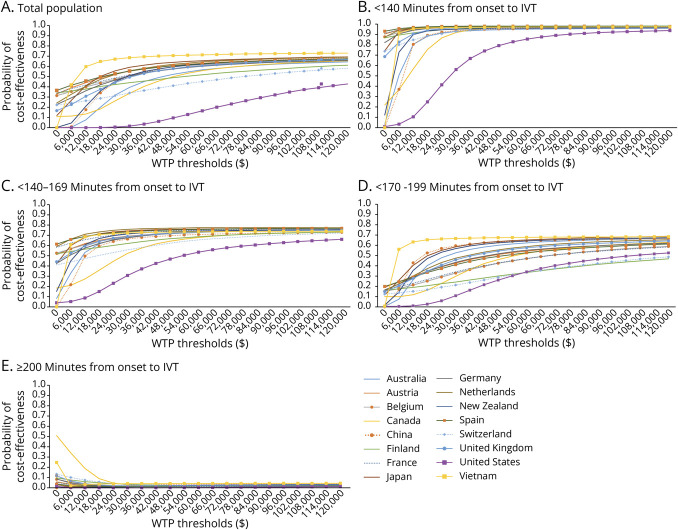
Cost-Effectiveness Acceptability Curves of IV Alteplase Plus EVT vs EVT Alone (A) Total population, not stratified by time from stroke onset to IVT; (B) <140 minutes from onset to IVT; (C) 140–169 minutes from onset to IVT; (D) 170–199 minutes from onset to IVT; (E) ≥200 minutes from onset to IVT. The y-axis shows the probability that IV alteplase plus EVT is cost-effective at different thresholds. EVT = endovascular thrombectomy; IVT = intravenous thrombolysis; WTP = willingness-to-pay.

One-way sensitivity analyses showed that the cost-effectiveness results changed substantially when varying health-related quality-of-life weights associated with mRS scores of 0–3 and the costs of IVT (eFigures 1–16). Probabilistic sensitivity analyses demonstrated that the probability of IV alteplase plus EVT being cost-effective varied across different onset-to-treatment time categories and WTP thresholds ranging from $0 to $120,000 (Figure 3). In the base-case analysis, which did not account for onset-to-IVT time, the probability of cost-effectiveness ranged from 19% to 65% at a WTP threshold of one GDP per capita. When onset-to-IVT time was less than 140 minutes, the probability of IV alteplase plus EVT being cost-effective was considerably higher: 82%–98%. For treatment initiated between 140 and 169 minutes, the probability declined to 51%–77% at the same WTP threshold. When onset-to-IVT time increased to 170–200 minutes, IV alteplase plus EVT was no longer cost-effective in several countries, with the probability of cost-effectiveness dropping to 32%–63%. Beyond 200 minutes, IV alteplase plus EVT consistently had a close to 0% probability of being cost-effective across all countries, regardless of the WTP thresholds.

## Discussion

This study adds to the existing literature by incorporating time-to-treatment in a cost-effectiveness analysis of IV alteplase plus EVT vs EVT alone in LVO stroke patients directly admitted to EVT-capable stroke centers. When onset-to-IVT time was not considered, IV alteplase plus EVT was cost-effective in 13 countries (50%–65% probability of being cost-effective) but not in the United States, China, and Vietnam. In the United States, this finding is largely attributable to the high cost of alteplase ($8,959), in contrast to other high-income countries where prices ranged from $1,014 to $2,859. In China and Vietnam, relatively low WTP thresholds ($12,614 and $4,347, respectively) explain initial IVT not being cost-effective, compared with thresholds ranging from $32,677 to $99,995 in other countries analyzed. Notably, overall health gains associated with IV alteplase were modest, with incremental QALYs ranging from 0.06 to 0.08 per patient across 16 countries. However, even small improvements can translate into substantial health benefits when applied across the large population of stroke patients eligible for reperfusion treatment.

Our findings demonstrate that the cost-effectiveness of IV alteplase plus EVT is highly dependent on time-to-treatment. We did not further subdivide time intervals within 140 minutes because previous evidence shows^2^ that the clinical benefit of IV alteplase plus EVT is statistically significant only up to this point, and IV alteplase plus EVT was already cost-effective at 140 minutes in all countries. Earlier treatment would only be expected to yield greater benefit at similar costs. When administered within 170 minutes of symptom onset, IV alteplase plus EVT still remained cost-effective in all countries, even when applying conservative WTP thresholds set at one GDP per capita.^14^ Of interest, the finding that IVT plus EVT remained cost-effective beyond the previously reported 140-minute clinical threshold highlights the importance of evaluating time-dependent effects from an economic perspective. Within the <140-minute window, QALYs gained ranged from 0.28 to 0.37, compared with 0.16–0.25 QALYs gained in the 140-169-minute window. The modest yet persistent clinical differences in the acute phase of stroke translated into long-term economic advantages, given the substantial lifetime costs of post-stroke disability. Notably, the incremental benefit of IVT decreased substantially with treatment delays. Once onset-to-IVT time exceeded 200 minutes, IV alteplase plus EVT was no longer cost-effective in any country. These results underscore the clinical and economic importance of minimizing delays in acute stroke workflows. While major trials, mostly conducted in high-income countries, achieved median onset-to randomization times of approximately 140 minutes, only 48% of stroke patients in low-income and lower-middle-income countries reach hospital care within 24 hours of stroke onset.^1,24^ These disparities emphasize the need for context-specific treatment decisions and general policy implications that consider time-to-treatment and local health care capacity.

Our comprehensive literature review identified 8 cost-effectiveness studies comparing IV alteplase plus EVT and EVT alone. Notably, these studies were limited to 4 countries (Canada, China, the Netherlands, and the United States), none explicitly modeled the interaction between time-to-treatment and economic value.^3-6,20-23^ While studies conducted in the United States consistently found that IVT before EVT was not cost-effective, findings from China have been sensitive to the choice of effectiveness data from various trials. One study suggested that for patients with LVO stroke eligible for immediate treatment, the cost-effectiveness of IV alteplase plus EVT remained uncertain in both China and Canada.^5^ It is important to note that our findings, which integrated onset-to-IVT time, offer clear evidence on the value of IVT plus EVT across different health system contexts. This approach provides insights into the conditional benefit of IVT when administered promptly. For example, even in the United States, where alteplase is considerably more expensive, the INMB reached $17,039 per patient when IVT was given within 140 minutes of symptom onset. As such, our study fills an important evidence gap by providing a time-dependent cost-effectiveness analysis based on patient-level data from 6 trials. These results both support and extend previous findings, showing that IVT plus EVT still offers high value for money but only when delivered within 170 minutes.

A recent cross-sectional survey of 282 stroke physicians showed that while most respondents still choose a combined IVT and EVT approach for patients with LVO, decision making varied significantly in more complex clinical scenarios.^25^ Notably, 40% of respondents reported being uncomfortable making treatment decisions in the face of uncertainty regarding IVT in stroke care for patients directly admitted to EVT-capable stroke centers. This finding aligns with previous work showing heterogeneity among physicians in their preferences for IVT+EVT vs EVT alone in mothership settings, as well as in the acceptable thresholds of uncertainty when comparing the 2 strategies.^26^ Such variation highlights the need for more clear evidence to guide treatment choices, particularly because registry data show that IVT use before EVT in LVO stroke patients directly admitted to EVT-capable centers is already declining.^27^ Therefore, our findings have immediate relevance for clinical practice and health care policies in stroke care. These findings provide valuable input for health technology assessment bodies and guideline committees in refining coverage decisions for IVT use in EVT-capable stroke centers. However, our results should not be extrapolated to patients receiving IVT in primary stroke centers who are subsequently transferred to comprehensive stroke centers for EVT under initial IVT coverage. In this drip-and-ship population, recent observational studies have reported associations between IVT before EVT and higher rates of recanalization and favorable functional outcomes.^28-30^ Because these data are based on nonrandomized studies, the observed associations should be interpreted with caution. Nevertheless, these patients may represent a subgroup in whom timely IVT could be relevant and the cost-effectiveness of IVT before transfer in this group should be examined in a separate analysis.

This study is an international cost-effectiveness analysis of reperfusion therapies that explicitly incorporates time-to-treatment effects, using individual patient-level data from 6 major trials. By integrating real-world cost estimates and life expectancy data, our analyses are applicable and generalizable across both high-income and middle-income countries. Furthermore, the model and input data can be adapted for use in other countries to support local decision-making and health care policies.

Still, this study has limitations. First, the use of averaged patient characteristics limited our ability to incorporate patient-level heterogeneity (i.e., age and comorbidities or imaging profiles) because of the aggregate nature of the cost-effectiveness model. For example, in clinical practice, neurologists may be less likely to choose combined IVT and EVT in patients older than 80 years, or those with concomitant dementia.^25^ In addition, we assumed that the major difference between treatment strategies lay in their effect on functional outcomes at 90 days and did not explicitly model rates of symptomatic intracranial hemorrhage. Although no significant time-by-treatment interaction was observed for hemorrhagic complications in a previous study, the rate of hemorrhage in the EVT alone group was slightly lower in the meta-analysis, potentially leading to deviations in both costs and effects in the IVT before EVT group.^2^ Third, potential delays introduced by the randomization procedure were not modeled. However, given the trial design and monitoring, these are unlikely to have had a major impact on treatment effect estimates. Fourth, the clinical effect was derived from 6 trials conducted across 15 countries and may not be fully generalizable to the United States or to countries not represented in IRIS. The analysis included only direct medical costs, excluding informal care and productivity losses, which have been shown to be substantial after stroke. Indirect costs such as informal care, lost productivity accounted for approximately 50% of total costs during the first and second years after stroke.^31-33^ Last, we modeled IVT using alteplase, while tenecteplase is increasingly used in clinical practice for LVO stroke. Although the European Stroke Organisation expedited recommendation on tenecteplase (0.25 mg/kg) over alteplase (0.9 mg/kg) in LVO stroke patients, evidence for superiority of IV tenecteplase over alteplase in patients directly presenting to EVT-capable stroke centers remains limited.^34^ For example, in the AcT trial, no difference was observed between tenecteplase and alteplase in LVO strokes, and time dependency of treatment effects seemed similar across the 2 agents.^35,36^ It is important to note that tenecteplase is currently more expensive than alteplase in many health systems, which may further affect cost-effectiveness considerations. The BRIDGE-TNK trial recently demonstrated improved outcomes for IV tenecteplase before EVT compared with EVT alone in China.^37^ When ongoing trials including BRIDGE TNK ACT and DIRECT-TNK are available, future cost-effectiveness studies should evaluate tenecteplase in similar frameworks, particularly in low and middle-income countries where generalizability remains uncertain.^38,39^

To conclude, the cost-effectiveness of IV alteplase plus EVT, in patients directly admitted to EVT-capable stroke centers, varies by country and is highly dependent on onset-to-IVT time. Our findings show that IV alteplase plus EVT is cost-effective when IVT is administered within 170 minutes of onset, with progressively diminishing economic and clinical value thereafter. Beyond 200 minutes, the addition of alteplase is no longer cost-effective in any country assessed. These results emphasize the critical importance of minimizing treatment delays and can inform reimbursement policies and national guidelines regarding the use of IV alteplase in acute ischemic stroke.

## Appendix Coinvestigators

**Table:**

Coinvestigators are listed at Neurology.org.

## Glossary

EVT: endovascular thrombectomy
GDP: gross domestic product
INMB: incremental net monetary benefit
IV: intravenous
IVT: intravenous thrombolysis
LVO: large vessel occlusion
mRS: modified Rankin Scale
QALYs: quality-adjusted life years
WTP: willingness-to-pay

## References

[1] Majoie CB, Cavalcante F, Gralla J, et al. Value of intravenous thrombolysis in endovascular treatment for large-vessel anterior circulation stroke: individual participant data meta-analysis of six randomised trials. Lancet. 2023;402(10406):965-974. doi:10.1016/S0140-6736(23)01142-X 37640037

[2] Kaesmacher J, Cavalcante F, Kappelhof M, et al. Time to treatment with intravenous thrombolysis before thrombectomy and functional outcomes in acute ischemic stroke: a meta-analysis. JAMA. 2024;331(9):764-777. doi:10.1001/jama.2024.0589 38324409 PMC10851137

[3] Morsi RZ, Zhang Y, Zhu M, et al. Endovascular thrombectomy with or without bridging thrombolysis in acute ischemic stroke: a cost-effectiveness analysis. Neuroepidemiology. 2024;58(1):47-56. doi:10.1159/000535796 38128500 PMC10857025

[4] Nguyen CP, Lahr MMH, van der Zee DJ, et al. Endovascular thrombectomy alone for large vessel occlusion: a cost-effectiveness evaluation based on meta-analyses. Stroke. 2024;55(10):2482-2491. doi:10.1161/STROKEAHA.124.047276 39129622

[5] Ye Z, Zhou T, Zhang M, et al. Cost-effectiveness of endovascular thrombectomy with alteplase versus endovascular thrombectomy alone for acute ischemic stroke secondary to large vessel occlusion. CMAJ Open. 2023;11(3):E443-E450. doi:10.9778/cmajo.20220096 PMC10205869 37192770

[6] Ma H, Zhou Y, Gao L, et al. Cost-effectiveness of thrombectomy alone versus alteplase before thrombectomy in acute ischemic stroke: results from the DIRECT-MT. J Neurosurg. 2023;139(3):678-686. doi:10.3171/2022.12.JNS221791 36790013

[7] Fischer U, Kaesmacher J, Strbian D, et al. Thrombectomy alone versus intravenous alteplase plus thrombectomy in patients with stroke: an open-label, blinded-outcome, randomised non-inferiority trial. Lancet. 2022;400(10346):104-115. doi:10.1016/S0140-6736(22)00537-2 35810756

[8] LeCouffe NE, Kappelhof M, Treurniet KM, et al. A randomized trial of intravenous alteplase before endovascular treatment for stroke. N Engl J Med. 2021;385(20):1833-1844. doi:10.1056/NEJMoa2107727 34758251

[9] Mitchell PJ, Yan B, Churilov L, et al. Endovascular thrombectomy versus standard bridging thrombolytic with endovascular thrombectomy within 4·5 h of stroke onset: an open-label, blinded-endpoint, randomised non-inferiority trial. Lancet. 2022;400(10346):116-125. doi:10.1016/S0140-6736(22)00564-5 35810757

[10] Suzuki K, Matsumaru Y, Takeuchi M, et al. Effect of mechanical thrombectomy without vs with intravenous thrombolysis on functional outcome among patients with acute ischemic stroke: the SKIP randomized clinical trial. JAMA. 2021;325(3):244-253. doi:10.1001/jama.2020.23522 33464334 PMC7816103

[11] Yang P, Zhang Y, Zhang L, et al. Endovascular thrombectomy with or without intravenous alteplase in acute stroke. N Engl J Med. 2020;382(21):1981-1993. doi:10.1056/NEJMoa2001123 32374959

[12] Zi W, Qiu Z, Li F, et al. Effect of endovascular treatment alone vs intravenous alteplase plus endovascular treatment on functional independence in patients with acute ischemic stroke: the DEVT randomized clinical trial. JAMA. 2021;325(3):234-243. doi:10.1001/jama.2020.23523 33464335 PMC7816099

[13] World Bank. GDP per capita [online]. August 20, 2024. data.worldbank.org/indicator/NY.GDP.PCAP.CD

[14] Iino H, Hashiguchi M, Hori S. Estimating the range of incremental cost-effectiveness thresholds for healthcare based on willingness to pay and GDP per capita: a systematic review. PLoS One. 2022;17(4):e0266934. doi:10.1371/journal.pone.0266934 35421181 PMC9009631

[15] Ganesh A, Luengo-Fernandez R, Wharton RM, et al. Time course of evolution of disability and cause-specific mortality after ischemic stroke: implications for trial design. J Am Heart Assoc. 2017;6:e005788. doi:10.1161/JAHA.117.005788 28603141 PMC5669183

[16] Rebchuk AD, O'Neill ZR, Szefer EK, Hill MD, Field TS. Health utility weighting of the modified rankin scale: a systematic review and meta-analysis. JAMA Netw Open. 2020;3(4):e203767. doi:10.1001/jamanetworkopen.2020.3767 32347948 PMC7191324

[17] Sanders GD, Neumann PJ, Basu A, et al. Recommendations for conduct, methodological practices, and reporting of cost-effectiveness analyses: Second panel on cost-effectiveness in health and medicine. JAMA. 2016;316(10):1093-1103. doi:10.1001/jama.2016.12195 27623463

[18] Husereau D, Drummond M, Augustovski F, et al. Consolidated health economic evaluation reporting standards (CHEERS) 2022 explanation and elaboration: a report of the ISPOR CHEERS II Good Practices Task Force. Value Health. 2022;25(1):10-31. doi:10.1016/j.jval.2021.10.008 35031088

[19] Vemer P, Corro Ramos I, van Voorn GA, Al MJ, Feenstra TL. AdViSHE: a validation-assessment tool of health-economic models for decision makers and model users. Pharmacoeconomics. 2016;34(4):349-361. doi:10.1007/s40273-015-0327-2 26660529 PMC4796331

[20] Ospel JM, McDonough R, Kunz WG, Goyal M. Is concurrent intravenous alteplase in patients undergoing endovascular treatment for large vessel occlusion stroke cost-effective even if the cost of alteplase is only US$1? J Neurointerv Surg. 2022;14(6):568-572. doi:10.1136/neurintsurg-2021-017817 34187871

[21] Han M, Qin Y, Tong X, et al. Cost-effective analysis of mechanical thrombectomy alone in the treatment of acute ischaemic stroke: a Markov modelling study. BMJ Open. 2022;12(4):e059098. doi:10.1136/bmjopen-2021-059098 PMC8987747 35387833

[22] Han L, Lan K, Kou D, et al. Cost-effectiveness of endovascular treatment for acute ischemic stroke in China: evidence from Shandong Peninsula. Health Econ Rev. 2024;14(1):37. doi:10.1186/s13561-024-00513-7 38836982 PMC11154974

[23] Qureshi AI, Akinci Y, Huang W, et al. Cost-effectiveness analysis of endovascular treatment with or without intravenous thrombolysis in acute ischemic stroke. J Neurosurg. 2023;138(1):223-232. doi:10.3171/2022.4.JNS22514 35901768

[24] Aguirre AO, Rogers JL, Reardon T, et al. Stroke management and outcomes in low-income and lower-middle-income countries: a meta-analysis of 8535 patients. J Neurosurg. 2023;139(4):1042-1051. doi:10.3171/2023.2.JNS222807 37856884

[25] Siddiqi AZ, Kashani N, Dmytriw AA, et al. Understanding physician preferences about combined thrombolysis and thrombectomy in patients with large vessel occlusion: an international cross-sectional survey. J Stroke Cerebrovasc Dis. 2024;33(12):108022. doi:10.1016/j.jstrokecerebrovasdis.2024.108022 39306059

[26] Kaesmacher J, Mujanovic A, Treurniet K, et al. Perceived acceptable uncertainty regarding comparability of endovascular treatment alone versus intravenous thrombolysis plus endovascular treatment. J Neurointerv Surg. 2023;15(3):227-232. doi:10.1136/neurintsurg-2022-018665 35232755 PMC9985721

[27] Schlemm L, Siebert E, Kleine JF, et al. Decline of thrombolysis rates before endovascular therapy in patients with acute anterior circulation large vessel occlusion ischemic stroke: a multicenter analysis from the German Stroke Registry. Eur Stroke J. 2023;8(3):610-617. doi:10.1177/23969873231177774 37243508 PMC10472953

[28] Seners P, Mlynash M, Ter Schiphorst A, et al. Intravenous thrombolysis use before inter-facility transfer for thrombectomy: association with efficacy and safety outcomes. Ann Neurol. 2025;98(4):871-880. doi:10.1002/ana.27303 40536361

[29] Seners P, Nehme N, Ter Schiphorst A, et al. Intravenous thrombolysis use in the late time window before interhospital transfer for thrombectomy. JAMA Neurol. 2026;83(1):60-67. doi:10.1001/jamaneurol.2025.4712 41324934 PMC12670263

[30] Liebart S, Lansberg MG, Adwane G, et al. Effectiveness and safety of IV thrombolysis before hospital transfer for thrombectomy in patients with basilar artery occlusion. Neurology. 2025;105(11):e214355. doi:10.1212/WNL.0000000000214355 41259723

[31] van Mastrigt G, van Heugten C, Visser-Meily A, Bremmers L, Evers S. Estimating the burden of stroke: two-year societal costs and generic health-related quality of life of the Restore4Stroke cohort. Int J Environ Res Public Health. 2022;19(17):11110. doi:10.3390/ijerph191711110 36078828 PMC9517815

[32] Lv W, Wang A, Wang Q, et al. One-year direct and indirect costs of ischaemic stroke in China. Stroke Vasc Neurol. 2024;9(4):380-389. doi:10.1136/svn-2023-002296 37788911 PMC11420908

[33] Barral M, Rabier H, Termoz A, et al. Patients' productivity losses and informal care costs related to ischemic stroke: a French population-based study. Eur J Neurol. 2021;28(2):548-557. doi:10.1111/ene.14585 33047452

[34] Alamowitch S, Turc G, Palaiodimou L, et al. European Stroke Organisation (ESO) expedited recommendation on tenecteplase for acute ischaemic stroke. Eur Stroke J. 2023;8(1):8-54. doi:10.1177/23969873221150022 37021186 PMC10069183

[35] Bala F, Singh N, Buck B, et al. Safety and efficacy of tenecteplase compared with alteplase in patients with large vessel occlusion stroke: a prespecified secondary analysis of the ACT randomized clinical trial. JAMA Neurol. 2023;80(8):824-832. doi:10.1001/jamaneurol.2023.2094 37428494 PMC10334294

[36] Singh N, Almekhlafi MA, Bala F, et al. Effect of time to thrombolysis on clinical outcomes in patients with acute ischemic stroke treated with tenecteplase compared to alteplase: analysis from the AcT randomized controlled trial. Stroke. 2023;54(11):2766-2775. doi:10.1161/STROKEAHA.123.044267 37800372

[37] Qiu Z, Li F, Sang H, et al. Intravenous tenecteplase before thrombectomy in stroke. N Engl J Med. 2025;393(2):139-150. doi:10.1056/NEJMoa2503867 40396577

[38] University of Calgary; Berry Consultants. ACT-GLOBAL Adaptive Platform Trial for Stroke. 2024. Accessed June 29, 2025. https://clinicaltrials.gov/study/NCT06352632

[39] Ministry of Health, Brazil; Boehringer Ingelheim; Medtronic. Randomization to Endovascular Treatment Alone or Preceded by Systemic Thrombolysis With Tenecteplase in Ischemic Stroke (DIRECT-TNK). 2022. Accessed June 29, 2025. https://clinicaltrials.gov/study/NCT05199194

